# An Unusual Complication of Intramedullary Pin Migration during Total Knee Arthroplasty – A Case Report

**DOI:** 10.1055/s-0041-1739173

**Published:** 2021-12-16

**Authors:** Sanjay Bhalchandra Londhe, Ravi Vinod Shah, Paras Banka

**Affiliations:** 1 Departamento de Ortopedia, Holy Spirit Hospital, Mumbai, Maharashtra, Índia; 2 Departamento de Ortopedia, Criticare Superspeciality Hospital, Mumbai, Maharashtra, Índia

**Keywords:** arthroplasty, replacement, knee, bone nails, intraoperative complications, orthopedic procedures

## Abstract

Total knee arthroplasty (TKA) is one of the most effective operations to relieve pain and improve function in the end stage of osteoarthritis (when bone on bone contact occurs). The intraoperative complications of TKA include fracture, tendon or ligament injury, and nerve or vascular complications. We herein describe an unusual complication of intramedullary pin migration within the femoral canal during TKA. A 72-year-old male patient underwent TKA with a posterior stabilization system with sacrifice of the posterior cruciate ligament. The distal femur was sectioned and balance was checked in extension. Then to make anterior, posterior, chamfer and notch cuts, the five-in-one anteroposterior (AP) cutting block was placed on the distal femur and the cuts were initiated. As there was a medial overhang of the cutting block, it was shifted laterally. While doing so, the pins had to be shifted too, and one of them was inadvertently hammered into the previously-created medullary canal opening of the femur. As usual orthopedic instruments, like the long straight artery forceps and pituitary rongeurs, failed to remove the migrated pin, an extralong laparoscopic grasper was used under fluoroscopy control to locate, grasp, and remove the migrated pin.

## Introduction

Total Knee Arthroplasty (TKA) is one of the most successful operations in orthopedics; it is highly effective in relieving pain and improving function. 1 The occurrence of complications during TKA impacts the postoperative outcome and the functional improvement of the patient. Pinaroli et al. 2 have described various intraoperative complications, which are mainly due to the surgical technique chosen, and they include periprosthetic fractures, 3 tendon or ligament injury, 4 and nerve 5 or vascular 6 complications. In the present article, we report a case of an unusual complication: pin migration into the femoral medullary canal during TKA. To the best of our knowledge, this complication has not been reported before.

## Case Report

**Fig. 1:**
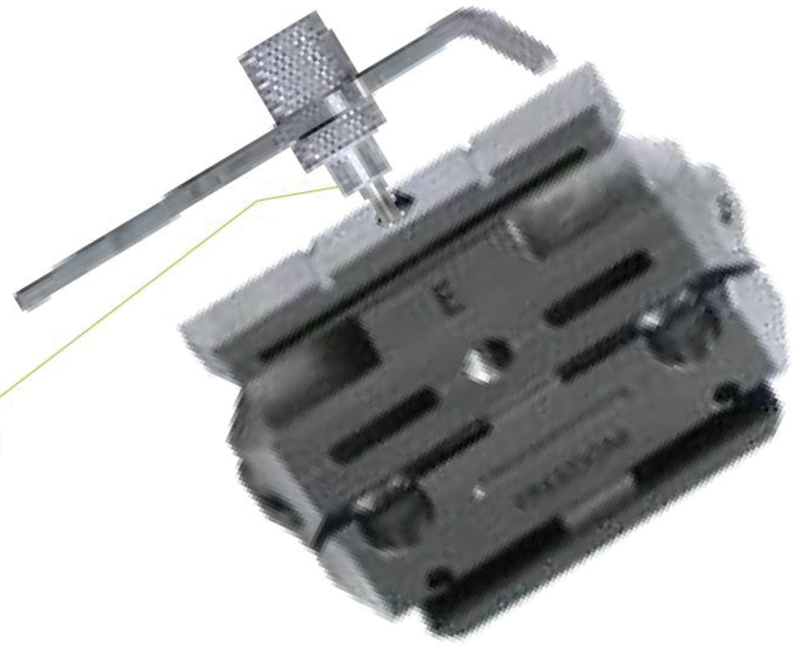
Five-in-one femoral cutting block.

**Fig. 2:**
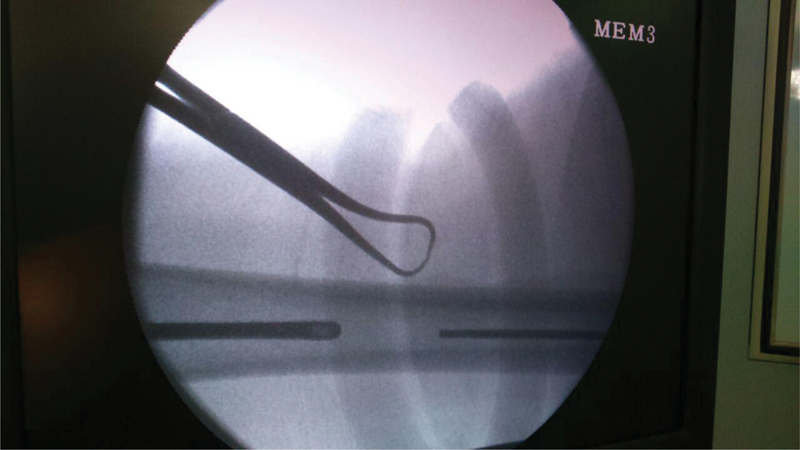
C-arm fluoroscopy image showing the proximal migration of the pin.

The patient was a 72-year-old male who complained of pain and difficulty in walking and climbing stairs for the previous 6 months. The pain increased considerably when squatting and sitting on low seats, and was relieved only partially with non-steroidal anti-inflammatory drugs (NSAIDs) and the local application of ice. Upon examination, the patient had a fixed flexion deformity of 15° with further flexion of 125° associated with crepitus and terminal movements, causing severe pain. Radiographs of the affected knee confirmed the clinical findings and showed advanced tricompartmental involvement, necessitating TKA. The patient was submitted to TKA under spinal anesthesia with the use of a tourniquet. A midline skin incision was performed with a medial parapatellar approach, and the joint was exposed, which reconfirmed the advanced tricompartmental involvement. A posterior stabilized knee system was used sacrificing the posterior cruciate ligament. The tibia was prepared first, followed by the femur. After the distal portion of the femur was cut and the gap was checked in extension, a five-in-one anteroposterior (AP) cutting block was placed and cuts were initiated. However, a medial overhang of the cutting block was noted, hence it was shifted laterally to prevent uneven condylar cuts. While doing so, the pins had to be shifted too, and one of them was inadvertently hammered into the previously-created medullary canal opening of the femur. Therefore, an attempt was made to retrieve the pin with the help of an artery forceps, which was in vain, as it resulted in the pin getting pushed further into the medullary canal. Then, pituitary rongeurs were used to circumvent the depth issue; this maneuver also failed, as we could not reach deep enough to hold on to the tip of the pin ( Figs. 1 and 2 ). With no success in sight, C-arm fluoroscopy was employed to visualize the exact position of the pin, which was far beyond the reach of the usual “grabbing” instruments. An attempt was made to even “drop” the leg down, using gravity in the hope that the pin would “fall down” the medullary canal. During this event, one of the operating team members suggested the use of a laparoscopic instrument to remove the pin.

**Fig. 3:**
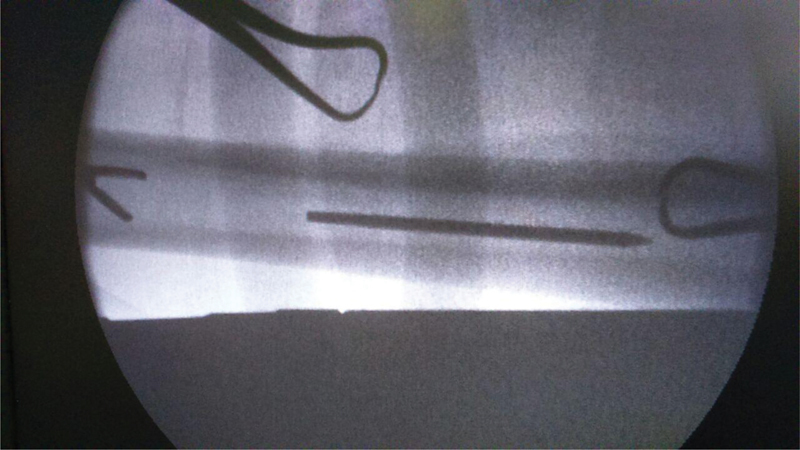
C-arm fluoroscopy image showing a pituitary rongeur falling short of the migrated pin.

**Fig. 4:**
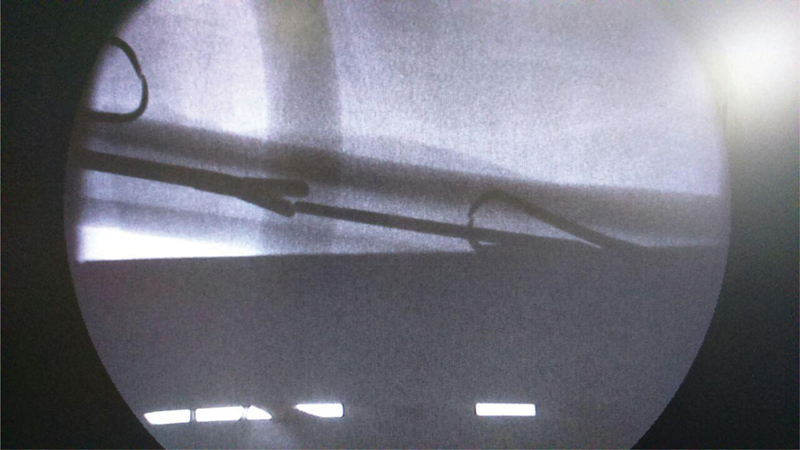
C-arm fluoroscopy image showing a laparoscopic instrument holding the migrated pin in the femoral medullary canal.

**Fig. 5:**
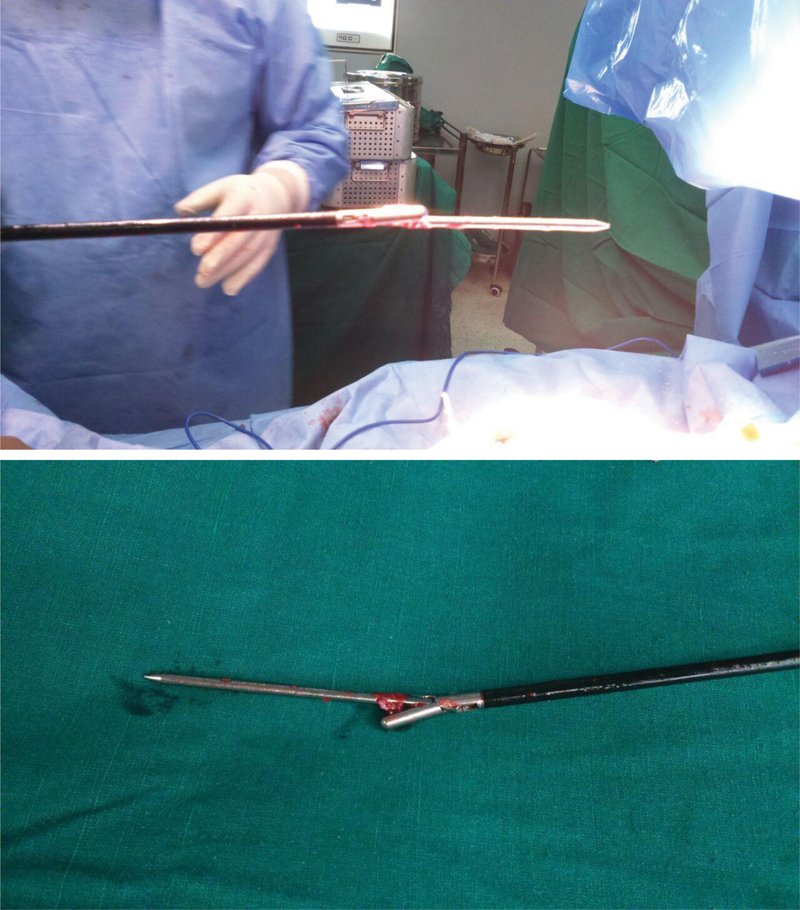
The retrieved migrated pin.

Fortunately, our operation theatre complex is well equipped with general and laparoscopic instruments. An extra-long laparoscopic grasper was used under fluoroscopy control to locate, grasp, and remove the migrated pin ( Figs. 3 , 4 and 5 ). Once the migrated pin was removed, the TKA procedure was performed in the usual manner. Postoperatively, the patient was informed about this intraoperative event. The postoperative course in the hospital was uneventful, and the patient made a very good functional recovery after the TKA.

## Discussion

Intraoperative complications can occur during TKA procedure. Pinaroli et al. 2 analyzed the intraoperative complications of 1,624 patients submitted to TKA, which included 69 fractures and ligament tears (3.8%), 40 fractures around the knee (2.2%), and 28 tendons or ligament tears (1.6%). In the study by Agarwala et al., 7 out of 3,168 primary TKAs performed between 2010 and 2017, 19 patients developed intraoperative fracture, 15 in the tibia and 4 in the femur, and most fractures occurred during cementing and final implantation (8 cases), followed by exposure and bone preparation (6 cases), and trialing (4 cases). One fracture occurred at an unknown time during the surgery. In the literature, there are many reports of pin-related complications with the use of computer-assisted navigation. 8 9 Beldame et al., 8 in a series of 385 TKAs, found an incidence of 1.3% (5 patients) of femoral fractures at the site of the tracker pin. Kamara et al. 9 reported a complication rate of 0.16% (n = 5) per pin site in a total of 3,136 pin sites in 839 patients. To the best of our knowledge, the present case report is the first in the literature which describes this unusual complication of pin migration to the femoral medullary canal. The operating surgeon needs to be very careful in placing the five-in-one femoral cutting block, so as to ensure that the block is placed in its designated place before initiating the cuts. Also, while pinning the five-in-one cutting block to the bone, the surgeon needs to be aware of the position of the open femoral medullary canal to prevent this avoidable error of migration of the pin.
