# Supplementary material for: An Unusual Complication of Intramedullary Pin Migration during Total Knee Arthroplasty – A Case Report
Source: Rev Bras Ortop (Sao Paulo). 2021 Dec 16;59(Suppl 1):e39–42. doi: 10.1055/s-0041-1739173 (PMC11254427; doi:10.1055/s-0041-1739173)
Supplement: Related texts

# Research article: Complicação incomum de migração do pino intramedular durante a artroplastia total de joelho – Relato de caso

**Keywords:** artroplastia do joelho, complicações intraoperatórias, pinos ortopédicos, procedimentos ortopédicos

## Abstract

A artroplastia total do joelho (ATJ) é uma das cirurgias mais eficazes para alívio da dor e melhora da função no estágio final da artrose (quando ocorre contato entre os ossos). As várias complicações intraoperatórias da ATJ incluem fratura, lesão em tendão ou ligamentos, e complicações nervosas ou vasculares. Neste artigo, descrevemos uma complicação incomum: a migração do pino intramedular dentro do canal femoral durante a ATJ. Um paciente do sexo masculino de 72 anos foi submetido a ATJ com sistema de estabilização posterior e sacrifício do ligamento cruzado posterior. A porção distal do fêmur foi seccionada, e o equilíbrio foi verificado em extensão. Em seguida, um bloco anteroposterior (AP) cinco em um foi utilizado para seção anterior, posterior, de chanfro, e entalhe. Por apresentar uma saliência medial, o bloco foi deslocado em sentido lateral. Ao fazê-lo, os pinos também tiveram de ser deslocados, e um deles foi inadvertidamente inserido na abertura do canal medular do fêmur criado. Como instrumentos ortopédicos usuais, como pinça reta longa e saca-bocado pituitário não conseguiram remover o pino migrado, uma pinça laparoscópica extralonga foi usada sob controle fluoroscópico para localizar, agarrar e remover o pino migrado.

## Introdução

Uma das cirurgias de maior sucesso em ortopedia, a artroplastia total do joelho (ATJ) é é altamente eficaz no alívio da dor e na melhora da função. ^1^ A ocorrência de complicações durante a ATJ afetam o desfecho pós-operatório e a melhora funcional do paciente. Pinaroli et al. ^2^ descreveram várias complicações intraoperatórias, que se devem principalmente à técnica cirúrgica escolhida, e elas incluem fraturas periprotéticas, ^3^ lesão em tendão ou ligamentos, ^4^ e complicações nervosas ^5^ ou vasculares. ^6^ Neste relato de caso, descrevemos uma complicação incomum: a migração do pino no canal medular femoral durante a ATJ. Pelo que sabemos, essa complicação ainda não foi relatada.

## Relato de Caso

Paciente do sexo masculino, de 72 anos, com queixa de dor e dificuldade para deambular e subir escadas havia 6 meses. A dor agravava-se especialmente ao se agachar e se sentar em lugares baixos, e era apenas parcialmente aliviada com anti-inflamatórios não esteroides e aplicação local de gelo. Ao exame, o paciente apresentava deformidade em flexão fixa de 15°, com flexão adicional de 125° associada a crepitação e movimentos terminais com dor intensa. As radiografias do joelho acometido confirmaram os achados clínicos e mostraram o envolvimento tricompartimental avançado e a necessidade de ATJ. O paciente foi submetido a ATJ sob raquianestesia com uso de torniquete. Uma incisão cutânea medial foi feita para a abordagem parapatelar medial; a articulação foi exposta, o que confirmou o acometimento tricompartimental avançado. Um sistema de estabilização posterior do joelho foi usado sacrificando o ligamento cruzado posterior. A tíbia foi preparada, seguida do fêmur. Após o corte da porção distal do fêmur e a verificação da lacuna em extensão, o bloco anteroposterior (AP) cinco em um foi colocado para o início das seções. No entanto, uma saliência medial do bloco de corte foi observada, portanto, ele foi deslocado lateralmente para evitar cortes condilares desiguais. Ao deslocar lateralmente o bloco, os pinos também tiveram que ser deslocados, e, nesse momento, um pino foi inserido de forma inadvertida na abertura do canal medular do fêmur criado. Uma tentativa de recuperação do pino com uma pinça reta e longa fracassou, e empurrou o material para o interior do canal medular. Em seguida, um saca-bocado pituitário foi utilizado para contornar o problema de profundidade, pois o pino havia migrado ainda mais para dentro do canal medular. Esta manobra, no entanto, também não teve sucesso, pois não conseguimos alcançar a profundidade suficiente para segurar a ponta do pino ( Figs. 1 e 2 ). Sem perspectiva de sucesso, um arco em C foi utilizado para a visualização da posição exata do pino, e mostrou que o material estava muito além do alcance dos instrumentos usuais de “agarrar”. Foi feita até mesmo uma tentativa de “soltar” a perna com a esperança de de que a gravidade fizesse o pino “cair” no canal medular. Nesse momento, um dos membros da equipe cirúrgica sugeriu o uso de um instrumento laparoscópico para a remoção do pino.

**Fig. 1:** Bloco femoral cinco em um.

**Fig. 2:** Imagem de fluoroscopia com arco em C mostrando a migração proximal do pino.

Felizmente, nosso centro cirúrgico é bem equipado com instrumentos gerais e laparoscópicos. Uma pinça laparoscópica extralonga foi usada sob controle fluoroscópico para localizar, agarrar e remover o pino migrado ( Figs. 3 , 4 e 5 ). Depois da remoção do pino migrado, a ATJ foi realizada da maneira usual. Após a cirurgia, o paciente foi informado sobre esse evento intraoperatório. A progressão pós-operatória do paciente no hospital transcorreu sem intercorrências, e a recuperação funcional após a ATJ foi muito boa.

**Fig. 3:** Imagem de fluoroscopia com arco em C mostrando que o saca-bocado pituitário não conseguia alcançar o pino migrado.

**Fig. 4:** Imagem de fluoroscopia com arco em C mostrando o instrumento de laparoscopia agarrando o pino migrado no canal medular do fêmur.

**Fig. 5:** Imagem do pino migrado recuperado.

## Discussão

Complicações intraoperatórias podem ocorrer durante o procedimento de ATJ. Pinaroli et al. ^2^ analisaram as complicações intraoperatórias de 1.624 pacientes submetidos a ATJ, que incluíam 69 fraturas e rupturas de ligamentos (3,8%), 40 fraturas adjacentes ao joelho (2,2%), e 28 rupturas de tendões ou ligamentos (1,6%). No estudo de Agarwala et al., ^7^ de 3.168 ATJs primárias realizadas entre 2010 e 2017, 19 pacientes sofreram fratura intraoperatória, 15 na tíbia e 4 no fêmur, e a maioria das fraturas ocorreu durante a cimentação e implante final (8 casos), seguidas de exposição e preparo ósseo (6 casos) e prototipagem (4 casos). Uma fratura ocorreu em momento desconhecido durante a cirurgia. Na literatura, há muitos relatos de complicações relacionadas a pinos de navegação computadorizada. 8 9 Beldame et al., 8 em uma série de 385 ATJs, observaram uma incidência de 1,3% (5 pacientes) de fraturas do fêmur no sítio do pino rastreador. Kamara et al. ^9^ relataram uma taxa de complicações de 0,16% (n = 5) por sítio de pino em um total de 3.136 sítios em 839 pacientes. Pelo que sabemos, este relato de caso é o primeiro na literatura que descreve essa complicação incomum de migração do pino no canal medular do fêmur. O cirurgião deve ter muito cuidado ao colocar o bloco cinco em um no fêmur, e se certificar da correspondência à porção distal do osso. Além disso, ao prender o bloco de corte cinco em um ao osso, o cirurgião precisa estar ciente da posição do canal medular do fêmur para evitar este erro de migração do pino.
